# PCNA-associated factor (KIAA0101/PCLAF) overexpression and gene copy number alterations in hepatocellular carcinoma tissues

**DOI:** 10.1186/s12885-021-07994-3

**Published:** 2021-03-20

**Authors:** Anchalee Tantiwetrueangdet, Ravat Panvichian, Pattana Sornmayura, Surasak Leelaudomlipi, Jill A. Macoska

**Affiliations:** 1 Research Center, Faculty of Medicine, Ramathibodi Hospital, Mahidol University, Bangkok, Thailand; 2 Department of Internal Medicine, Division of Medical Oncology, Faculty of Medicine, Ramathibodi Hospital, Mahidol University, Rama 6 Road, Rajthevi, Bangkok, 10400 Thailand; 3 Department of Pathology, Faculty of Medicine, Ramathibodi Hospital, Mahidol University, Bangkok, Thailand; 4 Department of Surgery, Faculty of Medicine, Ramathibodi Hospital, Mahidol University, Bangkok, Thailand; 5 Center for Personalized Cancer Therapy, University of Massachusetts Boston, Boston, MA USA

**Keywords:** PCNA-associated factor (KIAA0101/PCLAF), Droplet digital PCR, quantitative real-time PCR, p53 tumor suppressor protein, Ki-67 proliferation marker protein, HCC

## Abstract

**Background:**

PCNA-associated factor, the protein encoded by the KIAA0101/PCLAF gene, is a cell-cycle regulated oncoprotein that regulates DNA synthesis, maintenance of DNA methylation, and DNA-damage bypass, through the interaction with the human sliding clamp PCNA. KIAA0101/PCLAF is overexpressed in various cancers, including hepatocellular carcinoma (HCC). However, it remains unknown whether KIAA0101/PCLAF overexpression is coupled to gene amplification in HCC.

**Methods:**

KIAA0101/PCLAF mRNA expression levels were assessed by quantitative real-time PCR (qRT-PCR) in 40 pairs of snap-frozen HCC and matched-non-cancerous tissues. KIAA0101/PCLAF gene copy numbers were evaluated by droplet digital PCR (ddPCR) in 36 pairs of the tissues, and protein expression was detected by immunohistochemistry (IHC) in 81 pairs of formalin-fixed paraffin-embedded (FFPE) tissues. The KIAA0101/PCLAF gene copy number alteration and RNA expression was compared by Spearman correlation. The relationships between KIAA0101 protein expression and other clinicopathological parameters, including Ki-67, p53, and HBsAg protein expression in HCC tissues, were evaluated using Chi-square test.

**Results:**

Our results demonstrated that KIAA0101/PCLAF mRNA levels were significantly higher in HCC than in the matched-non-cancerous tissues (*p* < 0.0001). The high KIAA0101/PCLAF mRNA levels in HCC were associated with poor patient survival. The KIAA0101/PCLAF gene was not amplified in HCC, and KIAA0101/PCLAF gene copy numbers were not associated with KIAA0101/PCLAF transcript levels. KIAA0101 protein was overexpressed in the majority of HCC tissues (77.8%) but was not detectable in matched-non-cancerous tissues. Significant correlations between the expression of KIAA0101 protein in HCC tissues and p53 tumor suppressor protein (*p* = 0.002) and Ki-67 proliferation marker protein (*p* = 0.017) were found. However, KIAA0101 protein levels in HCC tissues were not correlated with patient age, tumor size, serum AFP level, or the HBsAg expression.

**Conclusions:**

KIAA0101/PCLAF mRNA and protein overexpression is frequently observed in HCC but without concurrent KIAA0101/PCLAF gene amplification. Significant correlations between the expression of KIAA0101 protein and p53 and Ki-67 proteins were observed in this study. Thus, detection of KIAA0101/PCLAF mRNA/protein might be used, along with the detection of p53 and Ki-67 proteins, as potential biomarkers to select candidate patients for further studies of novel HCC treatment related to these targets.

**Supplementary Information:**

The online version contains supplementary material available at 10.1186/s12885-021-07994-3.

## Background

Current global trends project hepatocellular carcinoma (HCC) to be the sixth most common cancer and the fourth leading cause of cancer-related deaths [1]. Therefore, identifying novel biomarkers for HCC diagnosis and prognosis could have great clinical utility and help improve patient outcomes. To this end, we applied a genome-wide cDNA microarray analysis and found that KIAA0101/PCLAF transcripts were significantly overexpressed in HCC tissues (Supplement Table S1). The KIAA0101/PCLAF gene was first cloned and sequenced from cDNA libraries of the immature myeloid leukemia cell line KG-1 [2]. PCNA-associate factor, the protein encoded by the KIAA0101/PCLAF gene, is an oncoprotein containing 111 amino acid residues with a conserved PCNA-binding motif (PIP-box, at amino acid residues 62–72), and also known as p15(PAF) or PAF15 [3], OEATC-1 (overexpressed in anaplastic thyroid carcinoma 1) [4], and L5 [5].

Functionally, the KIAA0101 protein (PCNA-associate factor) is a cell-cycle regulated oncoprotein that regulates DNA synthesis, maintenance of DNA methylation, and DNA-damage bypass, through its interaction with the human sliding clamp PCNA [3, 6–14]. KIAA0101 protein is part of the eukaryotic DNA-replication complex with PCNA, DNA polymerase delta (DNA Pol δ), and the endonuclease Fen-1 [6], and is primarily expressed during the S-phase of the cell cycle [7]. The Rb/E2F complex negatively regulates KIAA0101 gene expression; loss of Rb/E2F-mediated inhibition during the G1/S transition leads to upregulated KIAA0101 expression in the S-phase [7]. KIAA0101 protein levels drop rapidly at the mitotic exit (late M and G1 phases) via polyubiquitination mediated by the anaphase-promoting complex/cyclosome (APC/C) complex, a cell cycle-regulated E3 ubiquitin ligase, leading to its degradation by the proteasome [8]. The depletion of KIAA0101 significantly decreases DNA synthesis [6–9], indicating that KIAA0101 modulates the function of PCNA as a processivity factor [10, 11]. KIAA0101 is an intrinsically disordered protein that binds via its central PIP-box to the front-face of PCNA, and its N-terminus interacts with the inner ring of PCNA and passes through the PCNA from the back-face [10, 11]. UHRF1, an E3 ubiquitin ligase, ubiquitinates the N-terminal domain of KIAA0101 at Lys 15 and Lys 24 during the S-phase [9, 12]. Both the interaction of KIAA0101, via its PIP-box, with PCNA and the double mono-ubiquitylation at Lys 15 and Lys 24 by UHRF1 are required for KIAA0101 function in both DNA synthesis and maintenance of DNA methylation [9, 12–14]. Following UV-induced DNA damage, the resultant replication-fork-blocks trigger rapid, proteasome-dependent removal of Lys 15/24-ubiquitylated KIAA0101 from PCNA, which allows the interaction between monoubiquitinated PCNA and the translesion DNA synthesis (TLS) DNA polymerase eta (POLH) at stalled replisomes, thus facilitating the bypass of replication-fork-blocking lesions [9]. Following TLS-mediated damage bypass, the reassociation of KIAA010 with PCNA may help promote the dissociation of PCNA-associated TLS polymerases from PCNA and consequently resumption of normal replication [9].

KIAA0101 is overexpressed in various solid tumors [3–6, 15, 16], but few studies have directly investigated the expression of KIAA0101/PCLAF in hepatocellular carcinoma (HCC), and these studies have reported contradictory results [17–19]. Guo et al. reported down-regulation of KIAA0101 protein expression in HCC compared with non-cancerous liver tissues [17]. In contrast, Yuan et al. and Liu et al. reported overexpression of KIAA0101 at both mRNA and protein levels in HCC tissues [18, 19]. Gene amplification of cyclin D1, the regulatory component of the cyclin D1-CDK4 complex controlling the cell cycle G1/S transition, has been detected and implicated in HCC development and progression [20–22]. However, it remains unknown whether gene amplification of KIAA0101, the cell-cycle regulated oncoprotein, occurs or is causally correlated with KIAA0101/PCLAF overexpression in HCC. Droplet Digital PCR (ddPCR) enables high-throughput assessment of absolute gene copy number, compared with fluorescence in situ hybridization (FISH), while maintaining the sensitivity and precision [23]. Therefore, the present study aimed to correlate KIAA0101/PCLAF overexpression at the mRNA level, detected by quantitative real-time PCR (qRT-PCR), with KIAA0101/PCLAF gene copy number alterations detected by ddPCR in HCC tissues. This study also aimed to determine the relationships between KIAA0101 protein expression detected by IHC and other HCC clinicopathological parameters, including Ki-67, p53, and HBsAg protein expression in HCC tissues.

## Methods

### Tissue procurement

HCC and matched non-cancerous tissues, available from the same patients, were obtained by surgical resections from the operating rooms, Department of Surgery, Ramathibodi Hospital. Snap-frozen sections of resected liver tissues were prepared and stained with hematoxylin and eosin (H&E). The H&E stained histological slides were reviewed by an experienced pathologist. Samples were mapped with these H&E slides so that HCC and matched non-cancerous areas were selected for further studies.

### KIAA0101/PCLAF RNA expression level detection by quantitative real-time PCR (qRT-PCR)

KIAA0101 transcript levels were detected by multiplex qRT-PCR using qPCR primers specific for KIAA0101 tv1 (Roche Diagnostics, Germany). We have verified the specific pairing of the qPCR primers with KIAA0101 tv1 mRNA but not with KIAA0101 tv2 mRNA, as shown in Supplement Fig. S1. Total RNA from 40 pairs of HCC and matched non-cancerous tissues (Supplement Table S2 Cohort qRT-PCR) were extracted from snap-frozen samples using the RNeasy kit, including on-column DNase treatment (Qiagen, Germany) according to the manufacturer’s recommendation. The cDNA was synthesized with the iScript Advanced cDNA Synthesis Kit (Biorad, USA). LightCycler® 480 Probes Master (Roche, Germany) was used for performing multiplex qRT-PCR according to the manufacturer’s recommendation. The following primers and probe were used for specific KIAA0101 tv1 PCR: Forward primer 5’AGAAAGGTGCTTGGTTCTTCC3’; Reward primer 5’GGGTTCCCTCCTGCATATTT3’, and UPL probe no. 53. Human PBGD Gene of Universal Probe Library Reference Gene Assays (Roche, Germany) was used as an internal control. PCR was done in Light cycler 480 (Roche, Germany), with initial heating at 95^๐^C for 10 min followed by 40 cycles of 95^๐^C for 10 s, 60^๐^C for 30 s, 72^๐^C for 1 s, and cooling 40^๐^C for 30 s. Triplicate reactions were run for each sample. The expression level was calculated relative to the constitutive housekeeping gene PBGD using the 2^-∆∆CT^ method. The expression ratio (tumor/non-tumor) more than 2 was determined as KIAA0101 mRNA overexpression. The PCR reaction mixture with no cDNA template was used as a negative control.

The Cancer Genome Atlas (TCGA) data was interrogated for KIA0101/PCLAF and MKI67 transcript expression levels in relation to HCC patient survival, using tools available at https://www.proteinatlas.org.

### KIAA0101/PCLAF gene copy number alterations detected by droplet digital PCR (ddPCR)

DNA from 36 pairs of HCC and matched non-cancerous snap-frozen tissues (Supplement Table S3 Cohort ddPCR) were extracted by QIAamp DNA Mini Kit (Qiagen, Germany). DNA concentration was measured by Qubit™ dsDNA BR Assay (Invitrogen, Oregon USA). Copy number analysis was assessed by ddPCR according to the manufacturer’s recommendation (Bio-Rad, USA). Primer and probe for ddPCR™ copy number assay (Bio-Rad, USA): KIAA0101 (unique assay ID: dHsaCNS701823286) and AP3B1 (unique assay ID: dHsaCP100001) were used. The droplets were produced by a droplet generator of the QX200 Droplet Digital PCR system (Bio-Rad) and were then transferred to a 96-well PCR plate for amplification using the C1000 Touch Thermal Cycler (Bio-Rad) applying a thermal cycle beginning at 94 ^๐^C for 10 min, followed by 35 cycles of 94 ^๐^C for 30 s and 60 ^๐^C for 60 s, and a final cycle of 98 ^๐^C for 10 min. Subsequently, a droplet reader (Bio-Rad) was used to calculate the number of both positive and negative droplet events from each PCR reaction mixture. A PCR reaction mixture with no DNA template was used as a reference control for potential PCR contamination. Triplicate reactions were run for each sample. The ddPCR data were analyzed using the QuantaSoft analysis software (Bio-Rad), which calculates the total number of droplets (positive droplets plus negative droplets).

### KIAA0101, Ki-67, p53, and HBsAg protein expression detected by immunohistochemistry assay (IHC)

Protein expression of KIAA0101, as well as Ki-67, p53, and HBsAg, was determined by IHC in 81 pairs of HCC and matched non-cancerous tissues (Supplement Table S4 Cohort IHC). Tissues were fixed in 10% buffered formalin, processed, and embedded in paraffin. Serial 4-μm sections were cut and placed on superfrost® plus slides. Slides were deparaffinized in xylene and rehydrated through graded concentrations of ethanol to distilled water, and then processed with UltraVision Quanto Detection System (Labvision, USA). Antigen retrieval and primary antibodies incubation time shown in Supplement Table S5 Reagents. Slides were counterstained with hematoxylin and mounted in permanent mounting medium, and then scanned with the Pannoramic MIDI digital slide scanner (3DHISTECH, Hungary). Tissues with positive staining for the specific antibodies were used as positive controls, and tissues with the omission of the specific antibodies were used as negative controls. The positive and negative controls were included in every batch of IHC staining.

Ki-67 protein expression was assessed by visually estimated percentage of cells with positive staining in the entire lesion. Ki-67 positive was defined as Ki-67 protein expression with positive nuclei ≥10% of cells, as used in our previous study and others [24, 25]. The protein expression of KIAA0101, p53, and HBsAg was individually determined by IHC as negative or positive staining of tumor cells. The cut-off value for KIAA0101 protein positivity was a KIAA0101 staining with positive nuclei in ≥1% of cells, regardless of the staining intensity, as previously reported by Yuan et al. [18]. The cut-off value for p53 protein positivity was a p53 staining with positive nuclei in ≥1% of cells, regardless of the staining intensity, as we previously reported [24]. The cut-off value for HBsAg protein positivity was an HBsAg staining with positive cytoplasmic staining > 1 cell in each tissue section, as we previously reported [26].

### Serum hepatitis B surface antigen (HBsAg) assay

Chemiluminescent microparticle immunoassays (CMIA) for the qualitative detection of hepatitis B surface antigen (HBsAg) in serum from the patients were performed using ARCHITECT HBsAg Qualitative II assay with ARCHITECT *i* system equipment (Abbot Laboratories, Illinois, USA). Serum of 125 μl (μl) was used for each test. ARCHITECT HBsAg Qualitative II Controls (negative- and positive-controls) and Calibrators were used for quality control. The sample with the ratio of Sample Relative Light Unit/Cut-off Relative Light Unit (S/CO) < 1.000 was interpreted as nonreactive (negative). The sample with the ratio of S/CO > 1.000 was interpreted as reactive, which was then confirmed either if > 100 S/CO by the Alere Determine™ HBsAg Test, a rapid in vitro qualitative immunoassay (Abbot Laboratories, Illinois, USA), or if < 100 S/CO by VIDAS® HBs Ag Ultra test, an Enzyme-Linked Fluorescent Assay (ELFA), (bioMérieux S.A., Marcy-l’Étoile, France). When the sample with S/CO > 1.000 was reactive with either of the confirmatory tests, it was interpreted as HBsAg positive. The above strategy was based on WHO guidelines on hepatitis B and C testing. Geneva: World Health Organization; 2017. License: CC BY-NC-SA 3.0 IGO. ISBN: 978–92–4-154,998-1) [27].

### Serum alpha-fetoprotein (AFP) assay

Electrochemiluminescence immunoassays (ECLIA) for the in vitro quantitative determination of alpha-fetoprotein (AFP) in serum from the patients were performed using the AFP kit with a Cobas e601 analyzer (Roche Diagnostics Limited [27] GmbH, Mannheim, GM). Ten microliter (μl) serum is used for each test. The normal cut-off value of serum AFP is ≤7.2 ng/ml.

### Statistical analysis

Statistical analysis was performed with SPSS v.11.5 (SPSS Inc., Chicago, Illinois, USA) or GraphPad Prism 7 (version 7.03). For quantitative data, the comparison between the two groups was done using Wilcoxon signed rank test. Correlation between KIAA0101 gene copy number and KIAA0101 RNA expression was determined by Spearman nonparametric-correlation. Correlations between KIAA0101 protein expression and other clinicopathological parameters were determined by Chi-square test (x^2^ test). *P* < 0.05 was considered statistically significant.

## Results

### KIAA0101/PCLAF transcript is significantly overexpressed in HCC

**Fig. 1:**
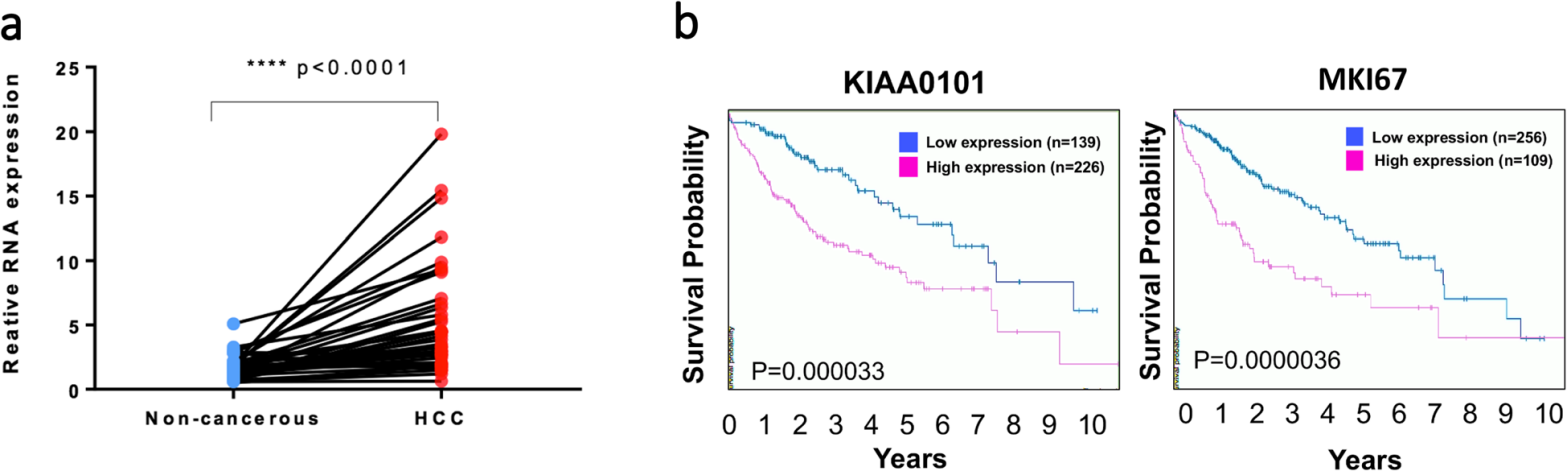
**a** Relative KIAA0101 RNA expression levels in 40 pairs of HCC and matched non-cancerous tissues were analysed by qRT-PCR. **b** TCGA data analysed for KIAA0101 AND MKI67 RNA expression in 365 HCC tissues and the patient survival

KIAA0101/PCLAF RNA expression levels were evaluated by qRT-PCR in 40 pairs of HCC and matched non-cancerous snap-frozen tissues. The mean relative RNA expression levels in HCC and matched non-cancerous tissues were 5.19 ± 4.31 and 1.67 ± 0.9, respectively. Significantly higher expression of KIAA0101 RNA in HCC tissues was observed (*p* < 0.0001) (Fig. 1a). This finding was confirmed by interrogating TCGA data, which also showed that KIAA0101/PCLAF and MKI67 transcripts were significantly upregulated in HCC. The high RNA expression levels of these genes in HCC were associated with poor patient survival: KIAA0101/PCLAF (*p* = 0.000033) and MKI67 (*p* = 0.0000036) (Fig. 1b). MKI67 is the gene encodes for the proliferation marker protein Ki-67.

### KIAA0101/PCLAF transcript levels are independent of gene copy number

**Fig. 2:**
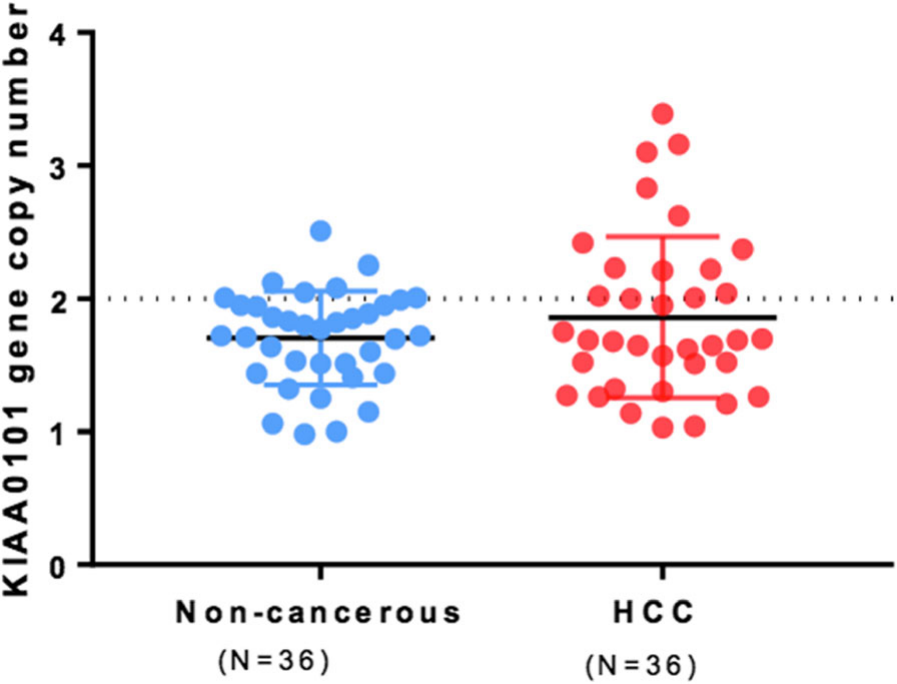
KIAA0101 gene copy numbers determined by ddPCR in HCC and matched non-cancerous tissues (*n* = 36)

**Fig. 3:**
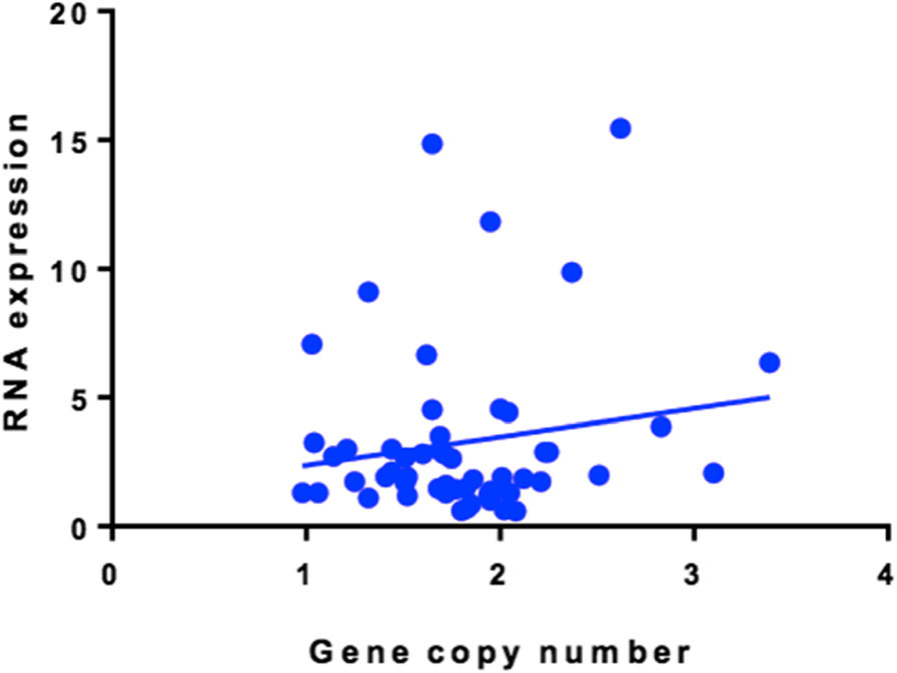
No correlation between KIAA0101 gene copy numbers and KIAA0101 RNA expression levels in 27 pairs of HCC and matched non-cancerous tissues (Spearman *r* = 0.02364)

KIAA0101 gene copy numbers were determined by ddPCR in 36 pairs of HCC and matched non-cancerous snap-frozen tissues. The results showed that KIAA0101 gene copy numbers were less than 4 copies and therefore not amplified in all HCC tissues. Consequently, KIAA0101 gene copy numbers in HCC and matched non-cancerous tissues were not significantly different (*p* = 0.24) (Fig. 2). Correlation between KIAA0101 gene copy numbers and KIAA0101 RNA expression levels was evaluated in 27 pairs of the snap-frozen tissues. However, no correlation was found between KIAA0101 gene copy numbers and KIAA0101 RNA expression levels (*r* = 0.002) (Fig. 3).

### Correlations of KIAA0101 protein overexpression and clinicopathological parameters

Eighty-one patients (69 men, 12 women; age range 23–94 years, median 56 years) resected for HCC were included in the KIAA0101 protein IHC study. The clinicopathological parameters of these patients are summarized in Table 1. Tumor size was ≥5 cm in 49% of patients. Serum HBsAg was detected in 64% of patients. Serum alpha-fetoprotein (AFP) values were ≥ 500 ng/ml in 23% of patients examined. The p53 and Ki-67 protein expression were detected in 43.2, and 46.9% of HCC tissues examined, respectively.

**Table 1:** Correlations between KIAA0101 overexpression in HCC tissues and other clinicopathological parameters (*n* = 81)

Variables	KIAA0101 overexpression^**a**^		P*
	Negative	Positive	
**Age**			
< 50	2 (9.1%)	20 (90.9%)	
> 50	16 (27.1%)	43 (68.2%)	0.07
**Tumor size**			
< 5 cm	9 (22%)	32 (78%)	
> 5 cm	9 (22.5%)	31 (77.5%)	0.953
**Serum AFP**			
< 500 ng/ml	12 (20%)	48 (80%)	
> 500 ng/ml	5 (26.3%)	14 (73.7%)	0.385
**HBsAg in HCC tissues**			
Negative	7 (24.1%)	22 (75.9%)	
Positive	11 (21.2%)	41 (78.8%)	0.757
**p53 protein expression**			
Negative	16 (34.8%)	30 (65.2%)	
Positive	2 (5.7%)	33 (94.3%)	0.002
**Ki-67 protein expression**			
Negative (< 10%)	14 (32.6%)	29 (67.4%)	
Positive (≥ 10%)	4 (10.5%)	34 (89.5%)	0.017

^a^Result given as n (%)

*Chi-square (χ^2^) test

**Fig. 4:**
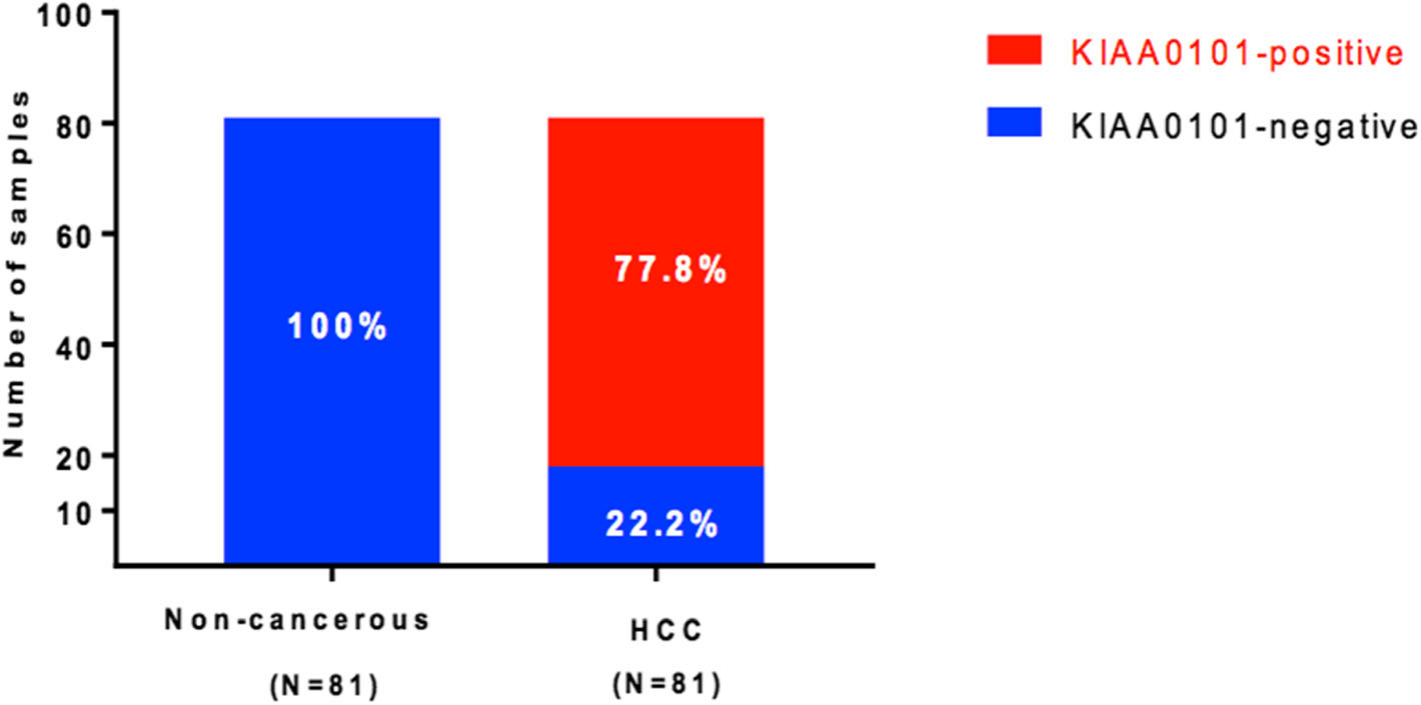
Summary of KIAA0101 protein expression detected by IHC as positive- and negative-staining in HCC and matched non-noncancerous tissues (*n* = 81)

**Fig. 5:**
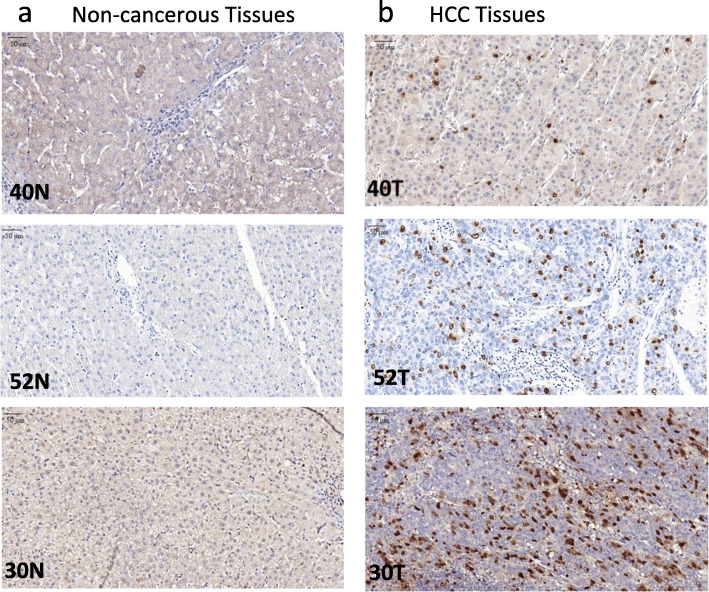
Representative of KIAA0101 protein expression: **a** KIAA0101 protein expression detected by IHC revealed negative staining in matched non-cancerous tissues. **b** KIAA0101 protein expression detected by IHC revealed positive nuclear staining in HCC tissues (magnification 40x)

**Fig. 6:**
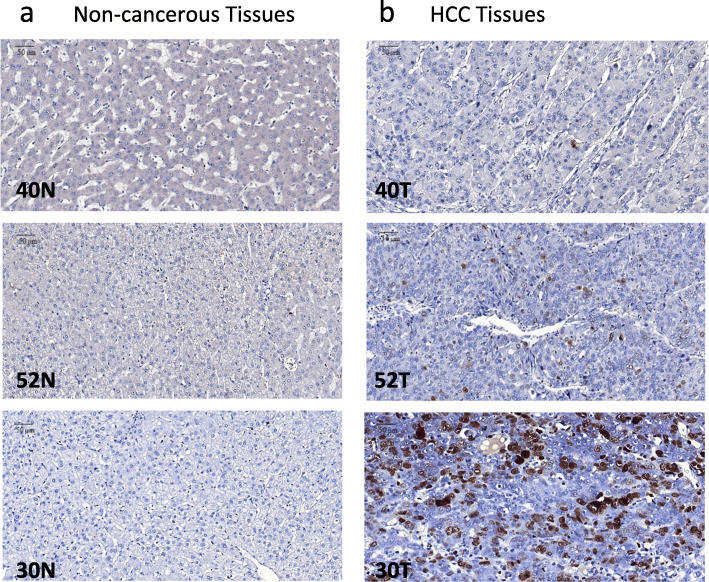
Representative Ki-67protein expression: **a** Ki-67 protein expression detected by IHC revealed negative staining in matched non-cancerous tissues. **b** Ki-67 protein expression detected by IHC revealed positive nuclear staining in HCC tissues (magnification 40x)

**Fig. 7:**
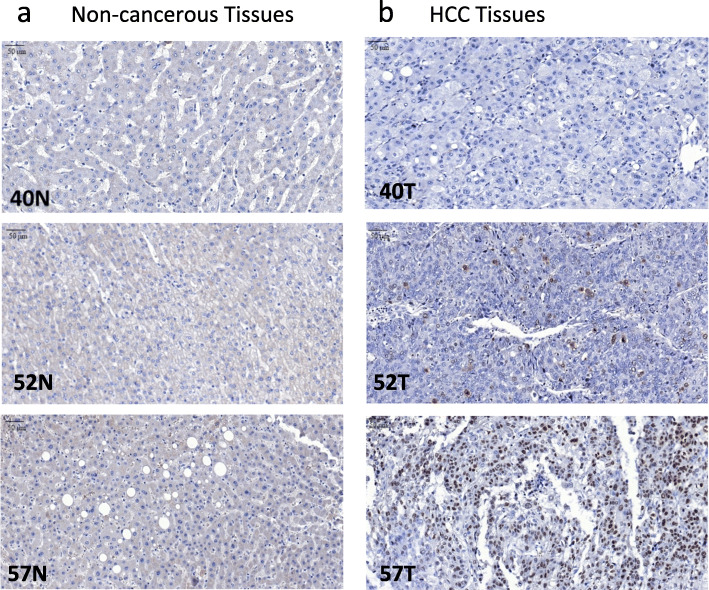
Representative p53 protein expression: **a** p53 protein expression detected by IHC revealed negative staining in matched non-cancerous tissues. **b** p53 protein expression detected by IHC revealed positive nuclear staining in HCC tissues (magnification 40x)

KIAA0101 protein expression was evaluated by IHC in 81 pairs of HCC and matched non-cancerous FFPE tissues. KIAA0101 protein was overexpressed, defined as positive nuclear staining, in 77.8% of HCC tissues examined but was not detectable, defined as negative nuclear staining, in 100% of non-cancerous tissues examined (Fig. 4). Representative KIAA0101 protein expression detected by IHC is shown in Fig. 5. Representative protein expression of Ki-67 and p53 detected by IHC is displayed in Figs. 6 and 7, respectively. These IHC images and statistical analysis illustrate that KIAA0101 protein expression in HCC was significantly correlated with p53 tumor suppressor protein (*p* = 0.002) and Ki-67 proliferation marker protein (*p* = 0.01), as shown in Table 1. However, no correlations were observed between KIAA0101 protein and age, tumor size, serum AFP level, and the HBsAg expression in HCC tissues.

## Discussion

PCNA-associated factor, the protein encoded by the KIAA0101/PCLAF gene, is a cell-cycle regulated oncoprotein that functions through the interaction with PCNA [3, 6–14]. KIAA0101 is overexpressed in various cancers [3–6, 15, 16]. However, few studies have directly investigated KIAA0101 expression in HCC, with contradictory results [17–19]. Furthermore, it remains unknown whether KIAA0101 overexpression is coupled to gene amplification in HCC. The present study aimed to investigate potential correlations between KIAA0101 mRNA overexpression, detected by quantitative real-time PCR (qRT-PCR), and KIAA0101 gene copy number alterations detected by ddPCR in our collected liver tissues. The study also aimed to investigate potential correlations between KIAA0101 protein expression, detected by IHC, and other clinicopathological parameters, including protein expression of Ki-67, p53, and HBsAg in HCC tissues.

In hepatocellular carcinoma (HCC), only a few studies have directly investigated the protein expression of KIAA0101, and interpretation of these studies are hampered by the use of different antibodies. First, as the antibody for KIAA0101 was not commercially available, Guo et al. prepared their own polyclonal rabbit antibody against a full-length KIAA0101-His tag, and they reported down-regulated KIAA0101 protein expression in HCC as compared with non-cancerous liver tissues [17]. Yuan et al. used semiquantitative reverse-transcription polymerase chain reaction (RT-PCR) for measurements of KIAA0101 mRNA, and a mouse monoclonal antibody against a full-length KIAA0101-GST tag (clone 3C11-1F11, Abnova) for immunohistochemical (IHC) analysis. They reported overexpression of KIAA0101 at both mRNA and protein levels in approximately 60% of HCCs, and found the association of KIAA0101 overexpression with higher tumor grade, higher tumor stage, and early tumor recurrence concordant with poor prognosis [18]. Yuan et al. also found a correlation of KIAA0101 overexpression with TP53 mutations; in their study, TP53 mutation occurred in approximately 50% of HCCs [18]. Lastly, Liu et al. reported that the KIAA0101 gene can be alternatively spliced to produce 2 transcriptional variants, which are translated to 2 protein variants: (1) KIAA0101 tv1 protein, the canonical sequence of 111 amino acid residues containing the PIP-box, and (2) KIAA0101 tv2 protein, the alternative sequence of 65 amino acid residues not containing the PIP-box [19, 28]. Liu et al. showed overexpression of KIAA0101 tv1 at both mRNA and protein levels in ~ 70% of HCC tissues (~ 40% in stage I-II, and ~ 80% in stage III-IV HCCs) as assessed by semiquantitative RT-PCR, virtual northern blot, western blot, and IHC analysis [19]. For IHC analysis, Liu et al. used a goat polyclonal antibody against a peptide mapping at the C-terminus of KIAA0101 (sc-65,163 antibody, Santa Cruz Biotechnology). In addition, Liu et al. found that doxorubicin (Adriamycin, ADR) treatment down-regulated the expression of KIAA0101 tv1, whereas the treatment increased the acetylation of p53 protein [19]. Recently, Liu et al. showed that KIAA0101 tv2 was highly expressed in adjacent non-tumorous liver tissues, compared to HCC tissues, and KIAA0101 tv2 could induce cell cycle arrest and apoptosis [28]. Using transfection, Liu et al. showed that KIAA0101 tv2 could inhibit the function of KIAA0101 tv1 by partially down-regulating KIAA0101 tv1, acting similar as a short hairpin RNA (shRNA) [28].

In our present study, KIAA0101 mRNA and protein expression levels were significantly higher in HCC than in the matched non-cancerous liver tissues, consistently with previous studies by Yuan et al. and Liu et al. [18, 19], but in contrast to Guo et al. [17]. We found no amplification of KIAA0101 gene in HCC, and no significant difference of KIAA0101 gene copy numbers in HCC and matched non-cancerous tissues, as well as no correlation between KIAA0101 gene copy numbers and KIAA0101 RNA expression. Our results indicate that KIAA0101 overexpression in HCC is not secondary to KIAA0101 gene amplification. Previous studies have suggested that KIAA0101 expression is regulated by the p53-p21 pathway [6], and the Rb/E2F pathway [7], as well as the NF-kB (p50) pathway [29], and the cAMP-dependent transcription factor ATF-3 [30]. Recently, Zhang et al. demonstrated that KIAA0101 is a direct transcriptional target of Forkhead box protein M1 (FoxM1), which may account for KIAA0101 overexpression in HCC [31].

This study also found significant correlations of KIAA0101 protein overexpression in HCC tissues with overexpression of p53 tumor suppressor protein and Ki-67 proliferation marker protein. The TP53 tumor suppressor gene, the most frequently mutated gene in cancer, encodes the p53 tumor suppressor protein [32, 33]. The wild-type p53 protein is a sequence-specific DNA-binding transcription factor, which upregulates many essential genes regulating various cellular processes, including cell cycle arrest, DNA repair, and cell death (apoptosis). The p53 protein has been known as the most important tumor suppressor and “the guardian of the genome” [34].

The p53 tumor suppressor protein is active as a tetramer, with four identical chains of 393 residues. Domain structure of the full-length p53 protein consists of an N-terminal transactivation domain, followed by a proline-rich region, the central DNA-binding domain responsible for sequence-specific DNA binding, the tetramerization domain regulating the oligomerization state of p53, and the extreme C-terminus [35]. According to Cancer Genome Atlas Research Network, the TP53 gene is inactivated by mutation in about 30% of HCC patients; the mutations are truncating mutations (frame-shift, nonsense, and splice-site mutations) in about one-third, and non-truncating mutations (missense) in about two-third. Most missense mutations of TP53 occur in the central DNA-binding domain and result in the defective p53 function [36].

The significant risk factors for HCC include chronic infections with the hepatitis B (HBV) or C (HCV) virus and exposure to dietary aflatoxin B1 (AFB1) or alcohol consumption. TP53 gene mutations occur in more than 50% of AFB1-induced HCC, in up to 45% of HBV-related HCC, and 13% of HCV-related HCC [37]. AFB1 frequently generates the hotspot TP53-R249S mutation and cooperates with HBV in causing TP53 mutations in HCC. Chronic HBV and HCV infection also induces reactive nitrogen/oxygen species that can damage DNA and mutate cancer-related genes such as TP53 [38]. HBx, the X gene of HBV, is the most common open reading frame integrated into the hepatic genome, and the integrated HBx is frequently mutated in HCC [38]. Mutant HBx proteins still retain their capability to bind to p53 protein and attenuate DNA repair and p53-mediated apoptosis. Thus, both viruses and chemicals are the causative agents of TP53 mutations during the molecular pathogenesis of HCC [38].

The p53 tumor suppressor protein is a transcription factor promoting cell-cycle arrest or apoptosis when cells encounter stress stimuli such as oncogene activation or DNA damage. The p53 protein is usually kept at low levels in unstressed cells by continuous ubiquitylation, primarily via the p53 protein interaction with the E3 ubiquitin-protein ligase Mdm2, then degradation by the 26S proteasome [39]. However, in stressed cells, p53 ubiquitylation is suppressed, and p53 protein is stabilized and accumulates in the nucleus, where it forms a tetramer. Only tetrameric p53 protein appears to be fully active as a transcriptional activator or repressor of distinct target genes containing p53 sequence-specific DNA binding sites. Furthermore, in stressed cells, p53 protein is subject to various post-translational modifications, including phosphorylation and acetylation, that influence p53 target genes’ expression. Phosphorylation and acetylation of p53 protein generally result in stabilization and accumulation of p53 protein in the nucleus, promoting transcriptional activation/repression of target genes. Missense-mutant p53 proteins generally show intense phosphorylation and acetylation at sites known to stabilize wild-type p53 protein. Thus, these post-translational modifications possibly facilitate the accumulation of dysfunctional mutant p53 in the nucleus [39].

The Cancer Genome Atlas (TCGA) has recently published the integrated analysis of the TP53 gene and pathway alterations in 32 cancer subtypes, including HCC [40]. More than 90% of TCGA cancers with TP53 mutations exhibit second allele loss by mutation, chromosomal deletion, or copy-neutral loss of heterozygosity (LOH). TP53 mutations are associated with enhanced chromosomal instability, including increased amplification of oncogenes (CCND1, CCNE1, ERBB2, and MYC) and deep deletion of tumor suppressor genes (RB1, PTEN, and WWOX) [40]. As compared with their non-mutated tumors, tumors with TP53 mutations display enhanced expression of cell cycle progression genes and proteins (cyclin B1, cyclin E1, FOXM1, and CDK1). Mutant TP53 cancers contained enhanced p53 protein expression, presumably the mutant p53 proteins derived from non-truncating (missense) TP53 mutations. Besides, proteins associated with the DNA damage response were also upregulated in mutant TP53 cancers [40]. In HCC, TP53 gene mutation is significantly correlated to p53 protein overexpression [41]. Consequently, p53 protein overexpression determined by IHC can predict TP53 gene mutations in HCC patients [42]. Therefore, the correlation between KIAA001 protein overexpression and p53 protein overexpression, found in this study, is consistent with the correlation between KIAA0101 overexpression and TP53 gene mutation in HCC as reported by Yuan et al. [18].

The Ki-67 proliferation marker protein is the nuclear antigen recognized by Ki-67 antibody; this antigen is present in proliferating cells but absent in resting cells [43]. The Ki-67 protein is a protein phosphatase 1 (PP1)-binding protein that organizes the mitotic chromosome periphery [44]. The Ki-67 protein acts as a biological surfactant and is required to maintain individual mitotic chromosomes dispersed in the cytoplasm following nuclear envelope disassembly [45]. The Ki-67 protein serves as the proliferation marker and is correlated with tumor growth rate and poor prognosis in HCC [46, 47]. Furthermore, KIAA0101 also serves as a proliferation marker and a poor prognosis marker in HCC [18]. Correlation between KIAA0101 and the Ki-67 protein overexpression has been reported in breast cancer by Kais Z et al. [48]. To the best of our knowledge, the present study is the first report that shows the correlation of KIAA0101 and the Ki-67 protein overexpression in HCC.

Our study shows the significant correlations of KIAA0101 protein overexpression with p53 protein and Ki-67 protein overexpression. Thus, it indicates that p53 protein accumulation and overexpression in HCC, most likely caused by non-truncating (missense) TP53 mutations [40], links with the enhanced cell cycle progression that requires the overexpression of KIAA0101 protein to cooperate with the PCNA for DNA synthesis and repair [10, 11]. The enhanced cell cycle progression also involves the overexpression of Ki-67 proliferation marker protein for maintaining individual mitotic chromosomes dispersed in the cytoplasm during the enhanced cell division [45].

Currently, the development of targeted cancer therapies are actively investigated against KIAA0101/p15PAF by agents with inhibitory functions similar to KIAA0101 tv2 [28], against p53 by anti-mutant p53 agents or MDM2/4 antagonists [49, 50], against Ki-67 by exploitation of MKI67 promoter to drive the expression of siRNAs or therapeutic genes [51], and against PCNA by inhibitors targeting to the modified PCNA involved in DNA repair [52]. These attempts might translate into novel therapeutics for HCC in the near future. Our study suggests that the candidates for these novel targeted therapies would be patients with KIAA0101 overexpression in their HCC tissues, which also correlated with p53 overexpression/mutation and Ki-67 overexpression.

## Conclusions

In summary, KIAA0101 overexpression, at mRNA and protein levels, frequently occurs in HCC without concurrent KIAA0101 gene amplification, indicating that KIAA0101 is probably transcriptional upregulated by other factors, such as FoxM1 or transcription factors in p53-p21 pathway, Rb/E2F pathway, etc. Significant correlations between the expression of KIAA0101 protein and p53 and Ki-67 proteins in HCC were observed in this study, indicating the connection between the aberrant p53 pathways and the enhanced cell cycle progression. Thus, detection of KIAA0101 mRNA/protein might be used, along with the detection of p53 and Ki-67 proteins, as potential biomarkers to select candidate patients for further studies of novel HCC treatment related to these targets.

## Supplementary Information

**Additional file 1: Table S1.** The top twenty differentially up-regulated genes in hepatocellular carcinoma tissues as compared to normal tissues are shown. KIAA0101 gene product is ranked in the 10th differentially up-regulated genes.

**Additional file 2: Supplement Table S2.** Cohort qRT-PCR.

**Additional file 3: Supplement Table S3.** Cohort ddPCR.

**Additional file 4: Supplement Table S4. **Cohort IHC.

**Additional file 5: Table S5.** Source, dilution, incubation time, conditions of different biomarkers.

**Additional file 6: Supplement Fig. S1. **The specific pairing of the qPCR primers with KIAA0101 tv1 mRNA but not with KIAA0101 tv2 mRNA.

## Data Availability

All data generated or analyzed during this study are included in this published article.

## Abbreviations

HCC: Hepatocellular carcinoma
PCNA: Proliferating cell nuclear antigen
KIAA0101: PCNA-associated factor (KIAA0101/PCLAF)
tv1: Transcriptional variant 1 (the canonical sequence)
tv2: Transcriptional variant 2
Rb/E2F complex: Retinoblastoma-associated protein/Transcription factor E2F complex
APC/C: Anaphase-promoting complex/cyclosome (APC/C) complex
ddPCR: Droplet digital PCR
qRT-PCR: quantitative real-time PCR
p53: p53 tumor suppressor protein
TP53: TP53 tumor suppressor gene
Ki-67: Ki-67 proliferation marker protein
MKI67: Gene of Ki-67 proliferation marker protein
