# Supplementary material for: PCNA-associated factor (KIAA0101/PCLAF) overexpression and gene copy number alterations in hepatocellular carcinoma tissues
Source: BMC Cancer. 2021 Mar 20;21:295. doi: 10.1186/s12885-021-07994-3 (PMC7981960; doi:10.1186/s12885-021-07994-3)
Supplement: Supplementary file 1 — Additional file 1: Table S1. The top twenty differentially up-regulated genes in hepatocellular carcinoma tissues as compared to normal tissues are shown. KIAA0101 gene product is ranked in the 10th differentially up-regulated genes. [file 12885_2021_7994_MOESM1_ESM.docx]

|  | **Gene Name** | **Gene ID** | **Score(d)** | **Numerator(r)** | **Denominator(s+s0)** | **Fold Change** | **q-value (%)** |
| --- | --- | --- | --- | --- | --- | --- | --- |
| **1** | **glypican 3** | **L47125.1** | **11.9356654598923** | **4.58863292056226** | **0.384447179420498** | **24.17331** | **0.737269282797264** |
| **2** | **serine protease inhibitor, Kazal type 1** | **NM_003122.1** | **7.91485066830905** | **3.23016271162985** | **0.408114170057987** | **9.24444** | **0.737269282797264** |
| **3** | **ribonucleotide reductase M2 polypeptide** | **NM_001034.1** | **5.42526807190887** | **2.96643785085** | **0.546781801660589** | **6.66733** | **1.6648016063164** |
| **4** | **ZW10 interactor** | **NM_007057.1** | **3.40197719054927** | **2.306309772163** | **0.677932167966896** | **6.62663** | **4.72628413919509** |
| **5** | **RA-regulated nuclear matrix-associated protein** | **NM_016448.1** | **4.73374930852579** | **2.43136497515176** | **0.513623518417571** | **6.32381** | **2.08035673595507** |
| **6** | **asp (abnormal spindle)-like, microcephaly associated (Drosophila)** | **NM_018123.1** | **4.52978056255719** | **2.68397287694441** | **0.592517195894634** | **5.86584** | **2.08035673595507** |
| **7** | **ubiquitin D** | **NM_006398.1** | **4.63380329014369** | **2.32331055341807** | **0.501383077343801** | **5.65533** | **2.08035673595507** |
| **8** | **thymidylate synthetase** | **NM_001071.1** | **3.64535456396946** | **2.31636167086543** | **0.635428359633454** | **5.58319** | **3.84295015185972** |
| **9** | **KIAA0186 gene product** | **NM_021067.1** | **4.54520270954244** | **2.330941521041** | **0.512835547718762** | **5.56857** | **2.08035673595507** |
| **10** | **KIAA0101 gene product** | **NM_014736.1** | **6.33833648405064** | **2.45582193112185** | **0.387455279047036** | **5.52674** | **1.6648016063164** |
| **11** | **CDC28 protein kinase regulatory subunit 2** | **NM_001827.1** | **3.42664906029224** | **2.24568149334652** | **0.655357888664267** | **5.45067** | **4.72628413919509** |
| **12** | **pyruvate kinase, muscle** | **NM_002654.1** | **4.04098068876239** | **2.25599987165646** | **0.558280290210294** | **5.38644** | **3.1705095437698** |
| **13** | **protein regulator of cytokinesis 1** | **NM_003981.1** | **4.20530084535809** | **2.42928531671237** | **0.577672182334796** | **5.31331** | **2.76583350362003** |
| **14** | **topoisomerase (DNA) II alpha 170kDa** | **NM_001067.1** | **3.66566859118346** | **2.48083493876107** | **0.676775566871454** | **5.02188** | **3.84295015185972** |
| **15** | **IGF-II mRNA-binding protein 3** | **NM_006547.1** | **3.43081797235719** | **2.04088539008017** | **0.594868456013697** | **4.9676** | **4.72628413919509** |
| **16** | **nucleolar protein ANKT** | **NM_016359.1** | **3.75823383107345** | **2.37969456720191** | **0.63319491925339** | **4.81978** | **3.84295015185972** |
| **17** | **MCM2 minichromosome maintenance deficient 2, mitotin (S. cerevisiae)** | **NM_004526.1** | **4.5569237712194** | **2.04409658496704** | **0.44856940506162** | **4.6307** | **2.08035673595507** |
| **18** | **Homo sapiens mRNA full length insert cDNA clone EUROIMAGE 1977059, mRNA sequence** | **NM_003107.1** | **3.9974112293071** | **2.23454968450846** | **0.558999201314543** | **4.55682** | **3.1705095437698** |
| **19** | **geminin, DNA replication inhibitor** | **NM_015895.1** | **5.61205009549167** | **2.36471723909741** | **0.421364242809782** | **4.55276** | **1.6648016063164** |
| **20** | **Rag D protein** | **AL138717** | **3.36525888352722** | **1.96521813661733** | **0.583972349419231** | **4.49796** | **4.72628413919509** |

**Supplementary Table S1 The top twenty differentially up-regulated genes in hepatocellular carcinoma tissues as compared to normal tissues are shown. KIAA0101 gene product is ranked in the 10^th^ differentially up-regulated genes.**

**This analysis is based on 8 Affymetrix HG-U133A GeneChips (4 hepatocellular carcinoma tissues and 4 non-tumor tissues) that were run** **and analyzed by Assist Prof. Jill Macosta in the Microarray core at the Comprehensive Cancer Center University** **Michigan for our group in early July 2003.**

**Gene Name – This is the name given by Affymetrix**

**Gene ID – This is the accession number. Score (d) – This is the t-statistic. The larger this value, the more significant the gene.**

**Numerator(r) – This is the numerator of the t-statistic**

**Denominator(s + s0) – This is the denominator of the t-statistic**

**Fold change – This is the fold change (in the Cancer tissue/Normal tissue)**

**q-value – This is the FDR equivalent of a p-value. It gives the percentage of the genes in the current row and above that are expected to be false positives.**
