# Supplementary material for: PCNA-associated factor (KIAA0101/PCLAF) overexpression and gene copy number alterations in hepatocellular carcinoma tissues
Source: BMC Cancer. 2021 Mar 20;21:295. doi: 10.1186/s12885-021-07994-3 (PMC7981960; doi:10.1186/s12885-021-07994-3)
Supplement: Supplementary file 2 — Additional file 2: Supplement Table S2. Cohort qRT-PCR. [file 12885_2021_7994_MOESM2_ESM.pdf]

No.	Case	age	Patho	AFP	tumor size	HBsAg_blood
1	1	≥50	HCC	60,500	19	NEG
2	2	<50	HCC grade 2	NEG	7	NEG
3	3	≥50	HCC grade 3	375.53	7	NEG
4	4	≥50	HCC	142.2	3.3	NEG
5	5	≥50	HCC	52	3	POS
6	6	<50	HCC grade 3	418	4	POS
7	7	≥50	HCC grade 3	25	10	POS
8	8	<50	HCC	12.66	9	POS
9	9	≥50	HCC	10,634	8	NEG
10	10	<50	HCC	3.7	10	POS
11	11	<50	HCC grade3	>40,000	8	POS
12	12	<50	HCC	261	4	POS
13	13	≥50	HCC	1,830	7	POS
14	14	<50	HCC	NEG	7	POS
15	15	≥50	HCC	Not done	5	NEG
16	16	<50	HCC	2,070	6	NEG
17	17	≥50	HCC	2	3	POS
18	18	≥50	HCC	343	7.6	POS
19	19	≥50	HCC	2,330	10	POS
20	20	<50	HCC	117	3.5	POS
21	21	≥50	HCC grade 2-3	Not done	Not done	Not done
22	22	≥50	HCC	17,072	5.5	NEG
23	23	≥50	HCC	23	1.5	POS
24	24	≥50	HCC grade 1-2	18	3	NEG
25	25	<50	HCC grade 3	17,900	3	POS
26	26	<50	HCC grade 3	Not done	5	POS
27	27	<50	HCC grade 2	8,312	9	POS

28	28	≥50	HCC	6,967	5	POS
29	29	≥50	HCC	4	10	POS
30	30	≥50	HCC	138	14	POS
31	31	<50	HCC	320	8	POS
32	32	<50	HCC	33,280	6	POS
33	33	≥50	HCC	14	2.2	POS
34	34	<50	HCC	26.3	3	POS
35	35	<50	HCC	390	5	POS
36	36	≥50	HCC	74.89	3	POS
37	37	<50	HCC grade 3	>40,000	9	NEG
38	38	<50	HCC	34	2	POS
39	39	≥50	HCC	3.5	16	NEG
40	40	≥50	HCC	Not done	Not done	Not done
