# Supplementary material for: PCNA-associated factor (KIAA0101/PCLAF) overexpression and gene copy number alterations in hepatocellular carcinoma tissues
Source: BMC Cancer. 2021 Mar 20;21:295. doi: 10.1186/s12885-021-07994-3 (PMC7981960; doi:10.1186/s12885-021-07994-3)
Supplement: Supplementary file 3 — Additional file 3: Supplement Table S3. Cohort ddPCR. [file 12885_2021_7994_MOESM3_ESM.pdf]

No.	Age	Patho	AFP	Tumor Size (cm)	HBsAg_blood
1	≥50	HCC	60,500	19	NEG
2	<50	HCC grade 2	NEG	7	NEG
3	≥50	HCC grade 3	375.53	7	NEG
4	≥50	HCC	3	1	POS
5	≥50	HCC	142.2	3.3	NEG
6	≥50	HCC	52	3	POS
7	≥50	HCC	242	3.5	POS
8	<50	HCC	418	4	POS
9	≥50	HCC	25	10	POS
10	≥50	HCC with vascular Invasion	23,640	8	POS
11	≥50	HCC	Not Done	8	NEG
12	<50	HCC and CIRRHOSIS	21	3	NEG
13	<50	HCC grade 3	>40,000	8	POS
14	<50	HCC	261	4	POS
15	≥50	HCC	ND	5	NEG
16	<50	HCC	2070	6	NEG
17	≥50	HCC	2	3	POS
18	≥50	HCC	343	7.6	POS
19	<50	HCC	1,780	Not done	POS

20	<50	HCC	117	3.5	POS
21	≥50	HCC	23	1.5	POS
22	≥50	HCC grade 1-2	18	3	NEG
23	<50	HCC	17,900	3	POS
24	<50	HCC	Not done	5	POS
25	<50	HCC	8,312	9	POS
26	≥50	HCC	138	14	POS
27	≥50	HCC	14	2.2	POS
28	<50	HCC	33,280	6	POS
29	<50	HCC	26.3	3	POS
30	<50	HCC	251	5	POS
31	<50	HCC	390	5	POS
32	≥50	HCC	74.89	3	POS
33	<50	HCC	34	2	POS
34	≥50	HCC	3.5	16	NEG
35	≥50	HCC	4.3	23.2	POS
36	<50	HCC	547	5	POS
