# Supplementary material for: PCNA-associated factor (KIAA0101/PCLAF) overexpression and gene copy number alterations in hepatocellular carcinoma tissues
Source: BMC Cancer. 2021 Mar 20;21:295. doi: 10.1186/s12885-021-07994-3 (PMC7981960; doi:10.1186/s12885-021-07994-3)
Supplement: Supplementary file 4 — Additional file 4: Supplement Table S4. Cohort IHC. [file 12885_2021_7994_MOESM4_ESM.pdf]

Case no.	Age	Tumor size	AFP	HBsAg	Ki-67	P53_T
1	≥ 50	3	5.5	pos	1	pos
2	≥ 50	7	62	neg	1	neg
3	≥ 50	4	2.9	pos	1	neg
4	≥ 50	2	4.2	pos	1	neg
5	≥ 50	4.5	193	pos	1	neg
6	≥ 50	3	393	pos	1	neg
7	≥ 50	2.8	1885	neg	1	neg
8	<50	4	79	pos	1	pos
9	≥ 50	8	2.9	neg	1	neg
10	≥ 50	4.2	38	neg	1	neg
11	≥ 50	2.2	2.2	pos	1	pos
12	≤ 50	13	60500	pos	1	pos
13	≥ 50	2	2	pos	1	neg
14	≥ 50	1.7	2	neg	1	pos
15	≥ 50	11.5	1219	neg	2	neg
16	≥ 50	2	2.9	pos	1	pos
17	≥ 50	5.5	1.5	neg	1	pos
18	≥ 50	7	279	pos	1	neg
19	≥ 50	2.5	4504	pos	1	neg
20	<50	7	60,000	pos	1	neg
21	≥ 50	1.7	4.7	pos	2	pos
22	≥ 50	4.5	946	neg	2	pos
23	≥ 50	3	2.1	pos	1	neg
24	≥ 50	4.5	17	pos	1	pos
25	≥ 50	3	41521	neg	1	neg
26	≥ 50	8	235	pos	1	neg
27	≥ 50	9	520	neg	1	neg
28	≥ 50	2.5	7.17	neg	1	pos

29	≥ 50	1.8	3.4	neg	1	pos
30	<50	16.7	6584	neg	1	neg
31	≥ 50	2.5	4.8	pos	1	pos
32	≥ 50	7	66	pos	1	neg
33	≥ 50	5	3.3	neg	1	neg
34	≥ 50	4.3	23	pos	1	neg
35	<50	16	18.6	pos	2	neg
36	≥ 50	4.5	17	pos	2	neg
37	≥ 50	2.5	1.8	neg	1	neg
38	≥ 50	6	7.3	pos	2	neg
39	≥ 50	10	61.3	neg	1	neg
40	≥ 50	8	5.1	pos	1	neg
41	<50	2	93.6	neg	2	pos
42	≥ 50	3	4.2	neg	2	neg
43	≥ 50	3.8	51	pos	2	pos
44	≥ 50	7	2.9	pos	2	neg
45	≥ 50	12	495	pos	2	pos
46	≥ 50	19	60,500	NEG	2	POS
47	<50	7	NEG	NEG	1	POS
48	≥ 50	7	375.53	NEG	2	POS
49	≥ 50	3.3	142.2	neg	2	NEG
50	≥ 50	3	52	POS	1	NEG
51	<50	4	418	POS	2	NEG
52	≥ 50	10	25	POS	2	POS
53	<50	9	12.66	POS	2	NEG
54	≥ 50	8	10,634	neg	1	neg
55	<50	10	3.7	pos	2	pos
56	≥ 50	4X6	32	NEG	2	POS
57	<50	8	>40,000	POS	2	NEG

58	<50	4	261	POS	2	NEG
59	≥ 50	5	2,566	pos	1	pos
60	≥ 50	5	ND	neg	2	NEG
61	≥ 50	3	2	pos	2	POS
62	≥ 50	7.6	343	pos	1	NEG
63	<50	3.5	117	POS	1	NEG
64	≥ 50	5.5	17,072	neg	2	NEG
65	≥ 50	1.5	23	POS	1	NEG
66	≥ 50	3	18	NEG	2	POS
67	<50	3	17,900	pos	2	POS
68	<50	5	ND	pos	1	NEG
69	<50	9	8,312	pos	2	NEG
70	≥ 50	5	6,967	POS	2	NEG
71	≥ 50	10	4	POS	2	NEG
72	≥ 50	2.2	14	pos	2	neg
73	<50	8	320	pos	2	NEG
74	<50	6	33,280	POS	2	POS
75	≥ 50	10	2,330	POS	2	POS
76	<50	3	26.3	POS	2	NEG
77	<50	5	251	pos	2	POS
78	<50	4.5	390	pos	2	POS
79	<50	2	34	POS	2	POS
80	≥ 50	16	3.5	NEG	1	NEG
81	≥ 50	2.8	417	neg	2	pos

Ki-67 : 1=<10%, 2= ≥ 10%
