# Supplementary material for: PCNA-associated factor (KIAA0101/PCLAF) overexpression and gene copy number alterations in hepatocellular carcinoma tissues
Source: BMC Cancer. 2021 Mar 20;21:295. doi: 10.1186/s12885-021-07994-3 (PMC7981960; doi:10.1186/s12885-021-07994-3)
Supplement: Supplementary file 5 — Additional file 5: Table S5. Source, dilution, incubation time, conditions of different biomarkers. [file 12885_2021_7994_MOESM5_ESM.docx]

**Supplement Table S5** Source, dilution, incubation time, conditions of different biomarkers

| Marker | Source | Host | Dilution | Antigen retrieval | Condition |
| --- | --- | --- | --- | --- | --- |
| KIAA0101 | 3C11-1F11 (Bio-Rad, formerly AbD Serotec) | Mouse | 1: 125 | 10 mM citrate buffer, pH 6.0 | 60 min, RT |
| P53 | Y5 (Lab vision, USA) | Rabbit | 1: 100 | 10 mM citrate buffer, pH 6.0 | 30 min, RT |
| Ki-67 | SP6 (Lab vision, USA) | Rabbit | 1: 200 | 10 mM citrate buffer, pH 6.0 | 30 min, RT |
|  |  |  |  |  |  |
| HBsAg | T9 (Lab vision, USA) | Mouse | 1: 200 | **-** | 60 min, RT |

RT= room temperature
