# Supplementary figures and images for: PCNA-associated factor (KIAA0101/PCLAF) overexpression and gene copy number alterations in hepatocellular carcinoma tissues
Source: BMC Cancer. 2021 Mar 20;21:295. doi: 10.1186/s12885-021-07994-3 (PMC7981960; doi:10.1186/s12885-021-07994-3)

## Slide 1
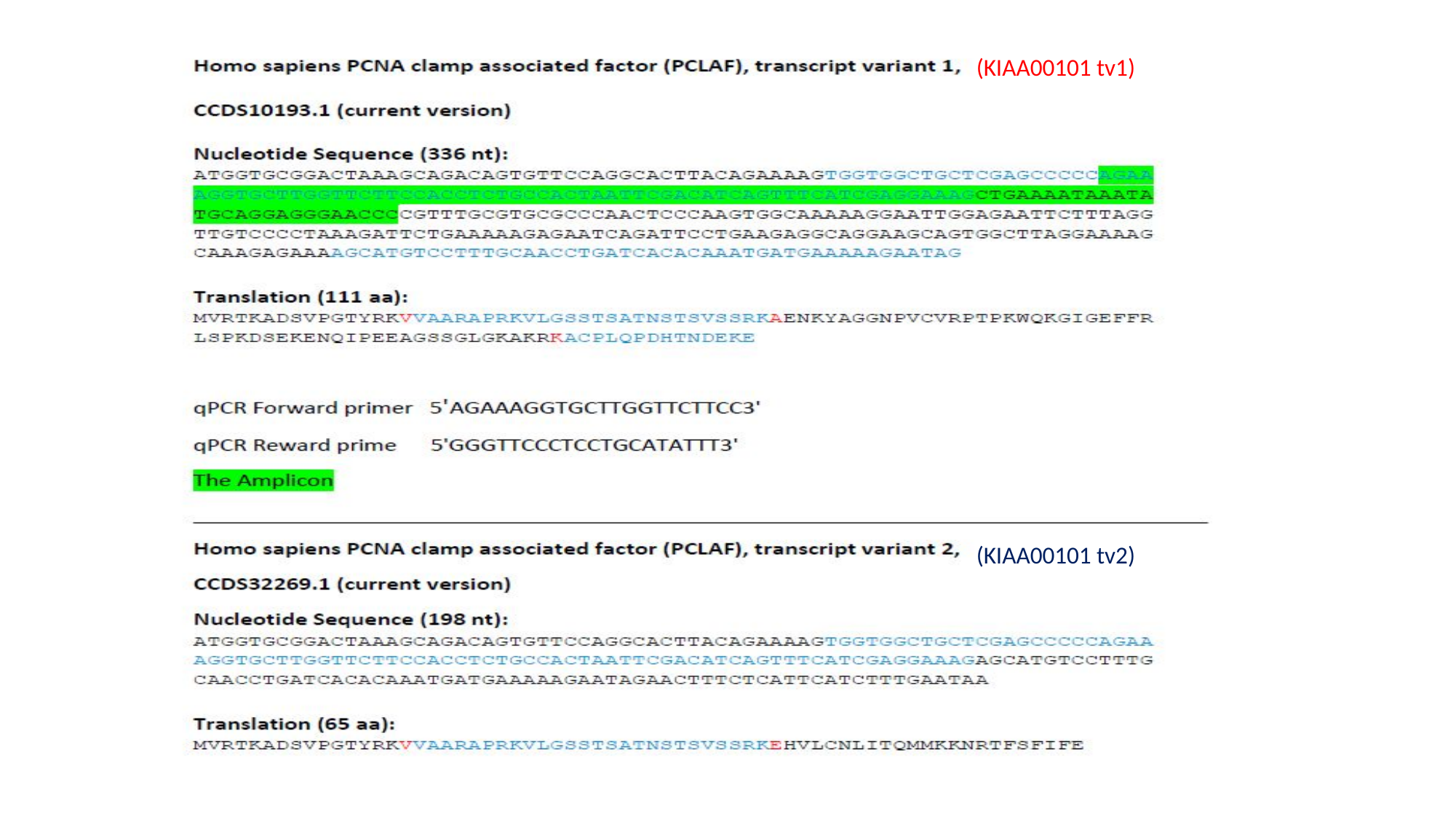

(KIAA00101 tv1)
(KIAA00101 tv2)

Supplement: Supplementary file 6 — Additional file 6: Supplement Fig. S1. The specific pairing of the qPCR primers with KIAA0101 tv1 mRNA but not with KIAA0101 tv2 mRNA. [file 12885_2021_7994_MOESM6_ESM.pptx]
